# Development and Performance Evaluation of an IoT-Integrated Breath Analyzer

**DOI:** 10.3390/ijerph20021319

**Published:** 2023-01-11

**Authors:** Abd Alghani Khamis, Aida Idris, Abdallah Abdellatif, Noor Ashikin Mohd Rom, Taha Khamis, Mohd Sayuti Ab Karim, Shamini Janasekaran, Rusdi Bin Abd Rashid

**Affiliations:** 1 Department of Mechanical Engineering, Faculty of Engineering, Universiti Malaya, Kuala Lumpur 50603, Malaysia; 2 Department of Management, Faculty of Business and Economics, Universiti Malaya, Kuala Lumpur 50603, Malaysia; 3 Department of Electrical Engineering, Faculty of Engineering, Universiti Malaya, Kuala Lumpur 50603, Malaysia; 4 Faculty of Management, Multimedia University, Cyberjaya 63100, Malaysia; 5 Center for Applied Biomechanics (CAB), Department of Biomedical Engineering, Faculty of Engineering, University of Malaya, Kuala Lumpur 50603, Malaysia; 6 Centre of Advanced Manufacturing and Material Processing (AMMP), Department of Mechanical Engineering, Faculty of Engineering, Universiti Malaya, Kuala Lumpur 50603, Malaysia; 7 Centre for Advanced Materials and Intelligent Manufacturing, Faculty of Engineering, Built Environment & IT, SEGi University Sdn Bhd, Petaling Jaya 47810, Malaysia; 8 Department of Psychological Medicine, Faculty of Medicine, Universiti Malaya, Kuala Lumpur 50603, Malaysia

**Keywords:** alcohol detection, breath analyzer, Internet of Things, HTTP, GSM, cellular IoT, linear regression, fuel-cell sensors, alcohol in breath

## Abstract

Although alcohol consumption may produce effects that can be beneficial or harmful, alcohol consumption prevails among communities around the globe. Additionally, alcohol consumption patterns may be associated with several factors among communities and individuals. Numerous technologies and methods are implemented to enhance the detection and tracking of alcohol consumption, such as vehicle-integrated and wearable devices. In this paper, we present a cellular-based Internet of Things (IoT) implementation in a breath analyzer to enable data collection from multiple users via a single device. Cellular technology using hypertext transfer protocol (HTTP) was implemented as an IoT gateway. IoT integration enabled the direct retrieval of information from a database relative to the device and direct upload of data from the device onto the database. A manually developed threshold algorithm was implemented to quantify alcohol concentrations within a range from 0 to 200 mcg/100 mL breath alcohol content using electrochemical reactions in a fuel-cell sensor. Two data collections were performed: one was used for the development of the model and was split into two sets for model development and on-machine validation, and another was used as an experimental verification test. An overall accuracy of 98.16% was achieved, and relative standard deviations within the range from 1.41% to 2.69% were achieved, indicating the reliable repeatability of the results. The implication of this paper is that the developed device (an IoT-integrated breath analyzer) may provide practical assistance for healthcare representatives and researchers when conducting studies involving the detection and data collection of alcohol consumption patterns.

## 1. Introduction

Alcohol consumption can vary between beneficial and harmful consumption. For example, correlations between occasional and daily drinkers and decreased loneliness, greater life satisfaction, and lower felt stress have been reported [1]. In contrast, high concentrations can lead to serious poisonous accidents [2]. Additionally, the harms of alcohol relative to the drinker may not only be demonstrated by the total volume of consumption but also by irregular heavy drinking [3,4].

Alcohol consumption prevails among individuals and communities for a variety of reasons. For instance, alcohol consumption could be a way to maintain a connection to tradition in some cultures [5], and it may be consumed due to social motives or mood/pleasure-enhancement motives [6]. According to the literature, alcohol consumption patterns can be associated with several community factors. For instance, in a previously documented systematic review by our team, it was revealed there is a strong correlation between alcohol consumption patterns and age, proximity to alcohol outlets, familial backgrounds, socioeconomic backgrounds, and religious influences [7]. Similarly, other recent records documented the social determinants of alcohol use and its consequences among communities [8,9,10,11,12,13,14].

The Internet of Things (IoT) envisions a future in which millions of things that act via sensing and actuation are linked and able to stream data over the Internet [15]. IoT entities allow smoother and faster data collection and effective physical world intervention. IoT enables cloud computing and overcome resources constrains challenges in multiple fields, including artificial intelligence [16], healthcare [17], and smart cities [18]. In healthcare, the automatic streaming of captured data via the IoT can instantly produce usable information from these data by sorting them into relevant categories and discovering associated patterns [19]. Additionally, IoT assistance in collecting health records is faster, more extensive, and constitutes more error-free data collection than manual approaches could possibly reach [20]. The functional framework of the IoT in healthcare may have different healthcare beneficiaries, including hospitals and community health centers [21]. Along with providing assistance in data collection, IoT has a promising future in providing diagnosis applications toward end users via web-based applications [22]. For example, IoT has been reported to be used in dermatological diagnosis [23], COVID-19 symptom diagnosis [24], predicting pathological conditions of cardiovascular diseases [25], and detecting and observing diabetes patients [26]. The communication technologies used for communications in IoT-integrated devices vary; along with the traditional methods such as WiFi, Bluetooth, and Ethernet, there are many other technologies such as radio-frequency identification and near-field communication, Bluetooth Low Energy, Li-Fi, ZigBee, Z-wave, and LoRa [27].

Breath alcohol content (BrAC) testing devices (breath analyzers) have been widely utilized as instruments for detecting alcohol (ethanol) in the human body. For instance, nearly every law enforcement agency is currently using breath analyzers [28]. Since blood alcohol content (BAC) levels are directly related to alcohol concentrations in our breath, estimating BAC via breath analysis is possible. However, the relationship of alcohol content in breath relative to that in the blood can be explained as the breath alcohol to blood alcohol ratio (BBR), and it may differ with different standards [29,30]. For instance, the units used for alcohol concentration measurements in the United States of America (USA) comprise grams of alcohol per 210 L of breath (g/210) for BrAC and grams of alcohol per 100 milliliters of blood (g/100 mL) for BAC, whereas the units used for alcohol concentration measurements in the United Kingdom (UK) comprise micrograms of alcohol per 100 milliliters of breath (mcg/100 mL) for BrAC and milligrams of alcohol per 100 milliliters of blood (mg/100 mL) for BAC [31,32]. In Malaysia, the measurement units used are mg/100 mL for BAC values and mcg/100 mL for BrAC values, and the BBR value is 2300 [31,32].

Breath analyzers have improved over the years, and further functionalities have been added. For instance, Previct Alcohol is a system for reporting BrAC; it is a personal pocket-sized breath analyzer connected to a smartphone that is further connected to a cloud database [33]. Hämäläinen et al. [34] used the personalized breath analyzer to introduce a new method for monitoring alcohol use disorder patients using an addiction monitoring index. Similarly, another pocket-sized breath analyzer is available for use with a smartphone, and it is called BACtrack. The device helped monitor and sustain responsible drinking [35], proved to be helpful in contingency management [36], and offers the possibility for further analytics [37].

In addition to breath analyzers, alcohol detection and tracking have been widely documented in the literature, and several technologies have been introduced. Furthermore, alcohol can be detected via wearable devices by utilizing the knowledge of alcohol metabolism in the body. For instance, sweat [38], tears [39], and interstitial fluids [40] have been used to detect alcohol in the human body.

One wearable technology that tracks alcohol concentration in sweat is the alcohol monitoring bracelet. For instance, the SCRAM alcohol monitoring bracelet is a wearable device used by law enforcement with law offenders to continuously monitor alcohol consumption by measuring transdermal alcohol concentrations [41]. Several studies researched alcohol monitoring bracelets. For instance, Lansdorp et al. [42] introduced a wearable band with a disposable cartridge using the enzyme alcohol oxidase to avoid the unreliability of degradable sensors. Lin et al. [43] reported successful detection and differentiation of ethyl clucuronide (a metabolite of alcohol formed in the body) concentrations in synthetic human sweat by correlating it with the predicted drinking profiles of moderate drinkers. Kinnamon et al. [44] demonstrated a device that uses square-wave voltammetry to detect ethyl glucuronide in human sweat, which allows understanding the impact of drinking on the individual in the long term.

Other wearable technologies for detecting alcohol in mediums other than sweat have also been documented. For instance, Sempionatto et al. [39] reported a wearable biosensor based on using eyeglasses, and the device collects tears and analyses them. Mohan et al. [40] described a wearable device for continuous alcohol monitoring using a microneedle for alcohol monitoring via human interstitial fluids. Thepchuay et al. [45] presented the “Blood Alcohol Micro-pad”, which offers the possibility for on-sight alcohol detection in whole blood.

Further advancements, including the implementation of the IoT in wearable devices for alcohol monitoring have also been reported by researchers and include BACtrack Skyn, Smart Start, and Milo ION [41,45]. In another research study, Li et al. [46] developed a wearable device to monitor alcohol levels by the IoT continuously; the data collected by the device can be transferred into a gateway smartphone via Bluetooth Low Energy to upload the data into a cloud for further analyses.

Detecting a person’s intoxication may be possible via virtual contactless methods. For example, Lamudomchai et al. [47] developed deep learning technology based on an infrared camera to analyze pictures taken based on stored data sets to identify the intoxication status of individuals.

Numerous alcohol detection and safety systems for helmets have been introduced in the literature. For instance, Maheswari et al. [48] introduced ethanol detection technology embedded into a smart helmet that allows the driver to use the vehicle under sober conditions. Midlaj Ali P et al. [49] proposed a smart helmet integrated with IoT to detect the intoxication of a driver in addition to providing an asleep alert. Tapadar et al. [50] developed an IoT-based helmet for motorbikes integrated with the ability to detect a falling helmet, possible accidents, the location of the rider, and the intoxication of the rider.

Other vehicle-related technologies have been presented as well. For instance, Anil Kumar et al. [51] introduced a black box that utilizes IoT technologies to detect alcohol and danger in the surroundings and act upon prepared actions such as identifying the location and alerting government representatives and emergency contacts. Nirosha et al. [52] developed a prototype of a device that senses alcohol in the atmosphere of the car and then reports to the nearest police station. Daw Khaing Zar Win [53] presented an alcohol detection system that can switch off vehicles upon sensing the presence of alcohol and report to an assigned phone number in case of an accident. Das et al. [54] developed a detection system for alcohol presence and the drowsiness of the driver using an eye-blink sensor, which provides further safety precautions. Wakana et al. [55] developed a detection system that can report the presence of alcohol and the presence of a human being in ambient surroundings by sensing the humidity of human breath. Ljungblad et al. [56] used real-time analysis to identify the position of the driver’s face and to simulate the direction of the exhaled air to run a breath analyzer accordingly. Manu et al. [57] developed an algorithm that can identify the driver’s eye position and its edges and analyze the state of drowsiness accordingly in addition to the detection of alcohol, and others implemented machine learning algorithms with alcohol sensing elements embedded in vehicles to provide even further enhanced alcohol detection [58,59].

Based on the literature review, we can categorize alcohol detection devices and technologies into five types: wearable devices, regular breath analyzers, personalized IoT-integrated breath analyzers, vehicle-integrated devices, and indirect detection technologies.

We used these categories of technologies to benchmark our proposed technology (Section 1.1), as shown in Table 1. The technologies’ abilities were explained by five criteria: portability, online connectivity, wide-scale usability, continuous monitoring, and independence. Portability includes the possibility of using the device’s full functionalities in different locations; online connectivity represents the implementation of IoT to connect the device to a cloud or database to stream any data; wide-scale usability defines the ability to implement the device across a community or a group of people without the need of assigning a single device for each individual; continuous monitoring is defined as the ability to record data in real time for a long period of time; and independence defines the ability of the device to connect to the cloud via a built-in gateway without the need of a third-party device, such as a smartphone.

Accuracy, processing time, and other technical measures of the results of these technologies are not included in the table as these criteria are objective and can vary across different implementations under the same category.

Wearable devices must be worn for long periods to allow the real-time tracking of alcohol levels (up to 24 h in legal situations); therefore, implementing these devices would compromise the comfortableness of the users and would require a single device for each individual. Regular breath analyzers are widely available and used by different institutions, including law enforcement institutions; however, the functions of these devices are often limited toward the detection and display of BrAC values without further functionalities. Personalized breath analyzers present an opportunity for individuals who are looking to track their alcohol use over time, and, due to their hand-held sizes, they are easily portable; however, implementing this technology would require a single unit for each user, which may present a challenge in covering a wide-scale study. Vehicle-integrated devices are devices that are integrated into vehicles or helmets; in addition to providing safety for traffic, they may be independent in terms of online connectivity as their power comes from vehicles, and unlike wearable devices, they have more freedom in terms of sizes. However, their usage is limited to vehicles rather than general applications. The indirect detection of alcohol intoxication has a promising future if implemented widely as it provides safety for public gathering places and workplaces without the time constraint of manual alcohol detection methods. To the best of our knowledge, no multi-user breath analyzer integrated with an independent cellular-IoT gateway technology and equipped with input (number pad) and output (LCD display) devices has been introduced in the literature.

### 1.1. Proposed Solution

In this paper, we propose an IoT-integrated breath analyzer based on the literature’s findings that indicate correlations between alcohol consumption and several social and demographic factors and based on the advantages of utilizing IoT in healthcare over manual methods. The developed IoT-integrated breath analyzer allows healthcare representatives to collect alcohol consumption data together with the identity of the consumer and instantly report these data into cloud databases, preserving time and effort and eliminating the possibility of errors in human reporting. Consequently, the collected information would reveal specific consumption patterns within a community or targeted individuals by identifying and associating different consumption patterns with related information, potentially enabling better-designed and tailored intervention programs.

### 1.2. Research Contribution

The requirements mentioned in the proposed solution in the previous section are to detect alcohol concentrations and communicate online with the cloud. The contributions of this work are summarized as follows:

1. An implementation of the hypertext transfer protocol (HTTP) to request and post participants’ information and breath-detected alcohol concentrations into a cloud database via cellular IoT technology.
2. Quantifying breath alcohol concentration by using a manually developed threshold and linear regression algorithm.
3. Performance evaluation of the developed alcohol quantification algorithm.

The remainder of this paper is structured as follows. Section 2 provides a brief background and history of breath analysis technology in detecting alcohol in breath and a brief background about the cellular IoT used in this study. Section 3 describes how the research study was conducted, introduces the developed framework, and explains how each functionality is developed. In Section 4, the results of the conducted experiments are shown, while Section 5 provides a discussion about the methodology and the results of this study. Finally, Section 6 and Section 7 conclude this paper and provide the outlook for future works related to the cellular IoT implementations in similar applications and the possible enhancements of this work.

## 2. Background

### 2.1. Breath Analyzers

The development of breath analyzers for quantifying alcohol concentrations in breath started in 1958 by Robert Frank Borkenstein with the implementation of a photometer coupled with potassium dichromate that reacts with ethanol in human breath [60]. Most breath analyzers currently implement similar concepts to those presented by Borkenstein by using sensing elements that contain an anode and a cathode to react with the ethanol and water present in the breath sample [61]; this can be referred to as an electrochemical or a fuel-cell sensor. Other sensing technologies have been used to detect alcohol in breath, including N-type semiconductors and infrared spectrometry [62]. N-type semiconductors have electrical resistance to air, which can be reduced with the existence of volatile compounds such as alcohol and, hence, possess the possibility of quantifying alcohol by relating the alcohol concentration to the change in resistance. However, other substances that exist in the human breath can affect these sensors, such as acetone [62]. Infrared spectrometry employs infrared spectroscopy to measure alcohol and may also utilize fuel-cell sensors. This technology can compare different parts of the supplied breath and can assess the stability of the alcohol concentration, but it is not portable [63].

During the breath test, fuel-cell sensors collect a breath sample that may contain compounds (ethanol molecules) in the platinum (Pt) anode compartment. This sample is drawn by a precise sampling pump that should be chosen to fit the fuel cell’s specifications. In the fuel-cell sensor circuit, the sensor does not require a power source; it produces an electric potential upon the oxidation reaction of ethanol in the sample. However, other components require a power source, including the microcontroller and the sampling pump. Due to the complex nature of ethanol oxidation reactions, various products can be generated, including acetaldehyde, acetic acid, and carbon dioxide [64], as shown in Reactions (1)–(3), respectively.

(1) $${\mathrm{CH}}_{3}{\mathrm{CH}}_{2}\mathrm{OH}\to {\mathrm{CH}}_{3}\mathrm{CHO}+2H^{+}+2e^{-}$$

(2) $${\mathrm{CH}}_{3}{\mathrm{CH}}_{2}\mathrm{OH}+H_{2}O\to {\mathrm{CH}}_{3}\mathrm{COOH}+4H^{+}+4e^{-}$$

(3) $${\mathrm{CH}}_{3}{\mathrm{CH}}_{2}\mathrm{OH}+H_{2}O\to {\mathrm{CO}}_{2}+12H^{+}+12e^{-}$$

**Figure 1:**
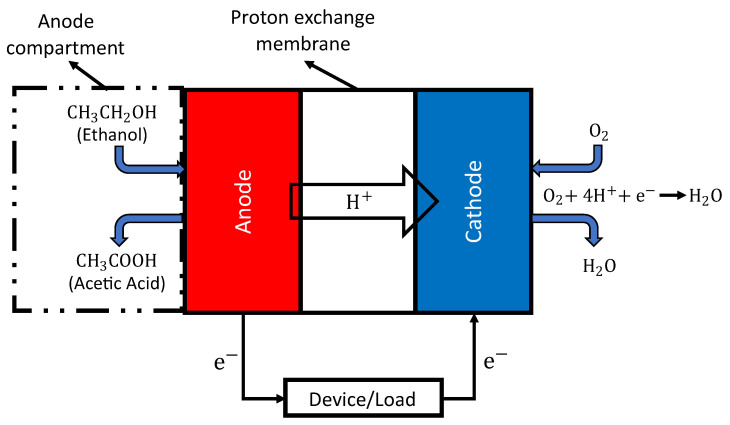
Schematic diagram of the fuel cell in breath analyzers.

However, the primary product of ethanol oxidation on Pt is acetic acid in the four-electron exchange process, as shown in Reaction (2) [65,66,67,68]. As shown in Figure 1, the electrolyte in the fuel cell is the proton exchange membrane; the electrons travel through the external load (the measurement device) as the membrane is insulated, and the protons permeate from the anode to the cathode through the proton exchange membrane.

The maximum open circuit potential (OCP) that a fuel cell can achieve can be provided by the Nernst equation based on the thermodynamic cell voltage [69,70]. Equation (4) calculates the thermodynamic voltage (*E*) under prevailing conditions, where $E^{o}$ is the reversible voltage standard at standard conditions at atmospheric pressure, *T* is the temperature, *R* is the ideal gas constant (8.314 $\frac{J}{K\cdot \mathrm{mol}}$ ), *n* is the number of transferred electrons in the reaction, *F* is the Faraday constant (96,485 $\frac{\mathrm{coulomb}}{\mathrm{mol}}$), $P_{r}$ is the partial pressure of the reactants, and $P_{p}$ is the partial pressure of the products.

(4) $$E=E^{o}+\frac{RT}{nF}\ln\frac{P_{r}}{P_{p}}$$

The Nernst equation predicts the OCP; however, fuel cells do not operate at the OCP. The actual voltage of a fuel cell is delivered after several losses have occurred; these losses include Ohmic losses, activation-related losses, and losses due to mass transparent limitations [69].

### 2.2. Cellular IoT and Internet Protocol

It is possible to connect physical objects (such as sensors and electrical devices) to the Internet by having them connected to the same networks as mobile phones; this is known as cellular IoT [71]. Cellular communication has the advantage of portability and usability in a wide range of locations depending on the coverage of the cellular operators in a country. With standards providing different data rates, the implementation of cellular connectivity can be chosen to fit the specific purpose it was chosen for. For instance, second-generation cellular network (2G) provides a data rate of less than 500 kilobits per second (kbps), third-generation cellular network (3G) provides a data rate of less than 2 megabits per seconds (Mbps), Long Term Evolution (LTE) provides a data rate of less than 10 Mbps, and fifth-generation cellular network (5G) provides the ability to transmit data with a rate up to 100 Mbps [27].

This research was conducted in Malaysia; Table 2 shows the available frequencies implemented by local cellular operators. The cellular connection can be made by using the proper type of chip depending on the available frequencies provided by the local operators. Some examples of subscriber identity modules (SIM) that can be integrated with microcontrollers are SIM900A, SIM700G, and SIM7600CE. SIM900A provides connectivity with the Global System for Mobile communication (GSM) interface only; SIM7000G is a version that offers the possibility to work with LTE CAT-M1 and NB-IoT (an enhanced version of LTE specifically for IoT); SIM7600CE works with the LTE standard as well as previous standards with several frequencies including B3, which is one of the frequencies available in Malaysia.

**Figure 2:**
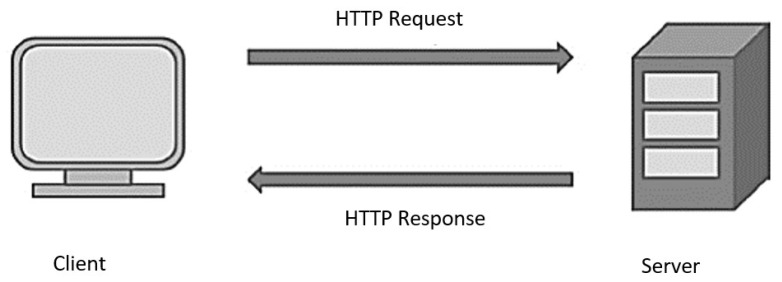
HTTP protocol.

Protocols, also referred to as communication protocols, are a collection of rules that enable devices to interact with one another. In communication, protocols define three points: syntax, semantics, and the synchronization of messages that are exchanged. Human languages are a similar analogy relative to protocols in terms of functionality. For Internet of Things applications, there are many communication protocols to choose from. HTTP, WebSocket, and Message Queuing Telemetry Transport (MQTT) are the most-used protocols, with HTTP being the most widely used [27]. HTTP allows the World Wide Web (WWW) to communicate with other computers on the Internet. In addition to being built on a client–server architecture, it works in a request-and-response manner (Figure 2). TCP (transmission control protocol) is used by HTTP to ensure that connections are reliable. HTTP is a stateless protocol, meaning that neither the client nor the server maintains a connection throughout the communication [27].

## 3. Methodology

**Figure 3:**
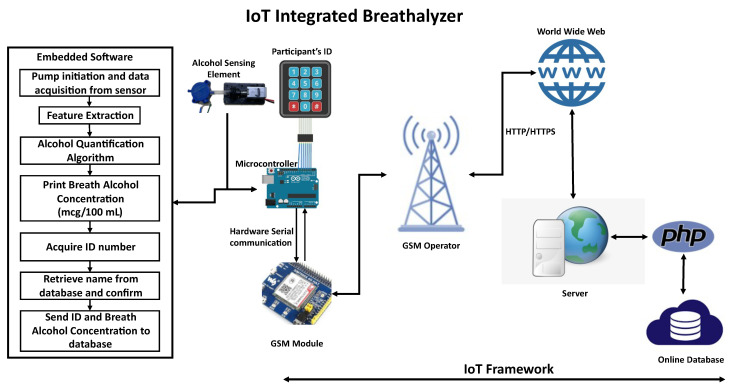
Overview of the developed framework.

This paper presents an IoT-integrated breath analyzer (Figure 3). The functionalities of this device can be divided into two main functions: IoT framework and breath analysis; however, several procedures were implemented throughout the entire development process. The following subsections provide the methodological approach followed by each process.

### 3.1. Conceptual Design

What we propose in this paper is a portable breath analyzer built with Malaysian standards and alcohol quantification units and integrated with an independent ability to communicate with an online database. Moreover, the device’s usage is not limited to a single user, including identity detection and confirmation via input (number pad) and output (LCD display) devices.

As previously explained (Section 2.1), fuel-cell sensors have great specificity to alcohol and insensitivity to substances such as acetone, which can prevail in the breath of people with diabetes. Therefore, the breath analyzer developed in this work implemented a 16 mm commercially available platinum fuel-cell sensor, an interference I/V-amplifying circuit, and a 0.35–0.4 milliliter (mL) sampling pump obtained from Dart Sensors Ltd. [73]. The output from the fuel cell was input to the amplifier, and the output from the amplifier was then passed through the analog-to-digital (A/D) converter in the electrical board used (Arduino Mega 2560) in order to be read by the microcontroller (ATmega 2560). The A/D converter displays readings by using voltage measurements. The reference voltage in the board during the data collection process was 4.94 volts (v). The A/D converter in the Arduino board maps the input voltages between 0v and the reference voltage in the board (4.94v) into integer values that are between 0 and 1023 [74]; hence, each unit of digital reading represents an increase of 4.82 mv. The sensitivity of the sensor is 13 mv/mcg/100 mL, and the maximum detectable concentration of the sensor according to the manufacturer is 300 mcg/100 mL BrAC.

**Figure 4:**
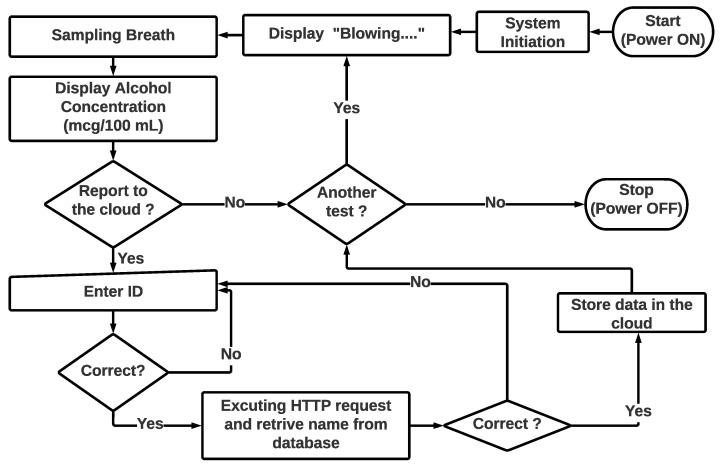
Conceptual design of the flow of the device’s function.

The IoT integration in this work enables online communication with online database for two purposes: identity confirmation with the database and uploading the detected breath alcohol concentration to a specific address in the database to be associated with the previously confirmed identity. Cellular technology was chosen as the communication technology due to its portability and broad coverage advantages. HTTP was used as a communication protocol due to the security provided by its stateless feature and the request–response nature of the proposed breath analyzer concept that resembles the HTTP nature. Figure 4 shows the conceptual design of the developed solution in a flow chart form to display the flow of embedded functionalities.

### 3.2. Data Collection

A wet bath standard was used via the data collection and experimental testing of the developed prototype with different ethanol concentrations within a range of [0, 200] mcg/100 mL and twenty trials for each concentration with a total of 200 samples. The concentrations used to develop the alcohol quantification algorithm were 0, 4, 10, 20, 30, 40, 50, 100, 150, and 200 mcg/100 mL. The sensor’s readings were sampled at 50 milliseconds (ms) intervals. The concentration was fed into the sampling chamber for 5 s, and the fuel-cell sampling pump was initiated to draw a breath sample of 0.35 mL and was released within the last 200 ms. After each trial, we waited until the sensor readings reached baseline before the next trial. The sensor readings were captured after the release of the sample as the sensor signal started peaking. The data collection for calibrations and evaluations was performed in the same laboratory using the same apparatus and data collection instruments.

**Figure 5:**
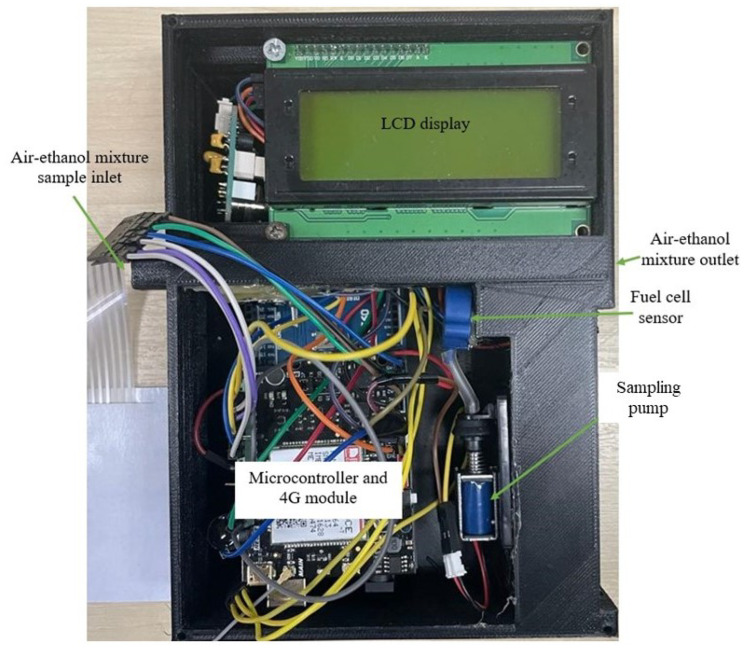
Designed casing for the testing and validation of the developed prototype.

**Figure 6:**
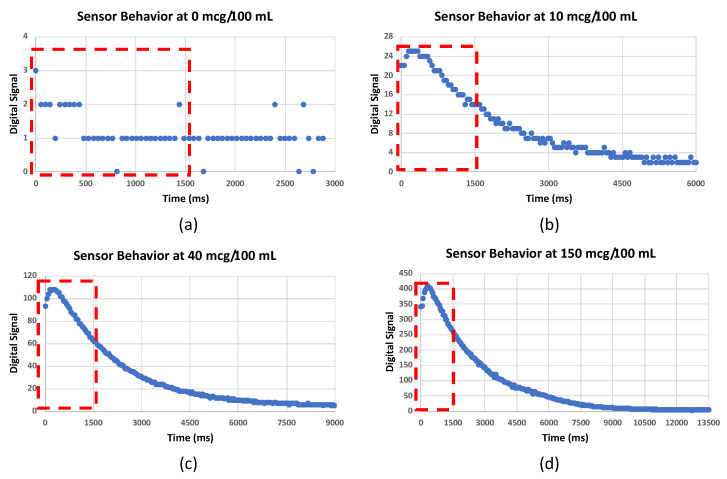
Output from alcohol sensors at 0 mcg/100mL (**a**), 10 mcg/100mL (**b**), 40 mcg/100mL (**c**), and 150 mcg/100mL (**d**); red dotted boxes show the area of interest used for feature extraction.

When calibrating the sensor, the emitted air sample must be presented to the sensing element in a controlled environment to avoid environmental noise. Figure 5 shows a 3D-printed design that contained the sensing element and the sampling pump; this design was used throughout all data collections. The breath sample should be passed through the air–ethanol mixture sample inlet, and the pump will draw a sample into the fuel cell from the sampling chamber (the pathway between the air–ethanol mixture inlet and outlet). Upon releasing the sampling pump, the fuel cell will immediately show a change in the digital output (within 200 ms); however, as higher concentrations are used, more time is needed to reach the peak, as shown in Figure 6.

### 3.3. Feature Extraction

The data collected as mentioned above (Section 3.2) were used to produce the alcohol quantification algorithm. The behavior of the sensor’s signal after the sampling was plotted into a graph (Figure 6) for observation. The first 30 readings of each sample (0 ms–1500 ms) were used as the area of interest for extracting features since they include sufficient information about the signal, including the peaking of the signal and the initiation of the downtrend toward the baseline.

We extracted features from each sample based on the area of interest, as shown in Figure 6. The features extracted are the mode of the first 10 readings (Mode10), as the readings after the first 10 readings tend to drop rather than remaining near the peak; the maximum value (Max30), which indicates the peak response of the sensor; the minimum value (Min30), which indicates the end point of the area of interest; the average of the first 20 readings (Avg20); the average of the first 30 readings (Avg30); the distance between the maximum value and the Avg20 (Max-Avg20); and the distance between the maximum value and the Avg30 (Max-Avg30). These features were used to develop the quantification algorithm (Section 3.4.1).

### 3.4. Alcohol Concentration Quantification Algorithm

In this section, we explain the method used to convert the raw digital readings of the sensor into concentrations measured in mcg/100 mL. The developed algorithm is then tested after being embedded into the microcontroller (Section 3.5).

#### 3.4.1. Threshold Algorithm

**Figure 7:**
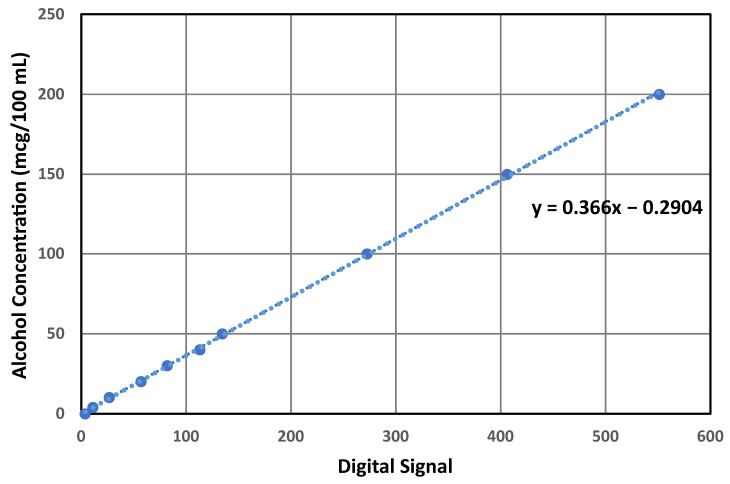
Linear equation obtained from plotting the average peaks of the digital readings (x) against alcohol concentrations (y).

It was observed that the sensor’s response exhibited linear (Figure 7) behavior. However, a single-point calibration may result in a biased performance relative to the particular calibration point. Therefore, the peak values obtained from previously collected data (Section 3.2) were averaged for each concentration and were used to obtain a linear relationship. Figure 7 shows the obtained linear equation.

**Figure 8:**
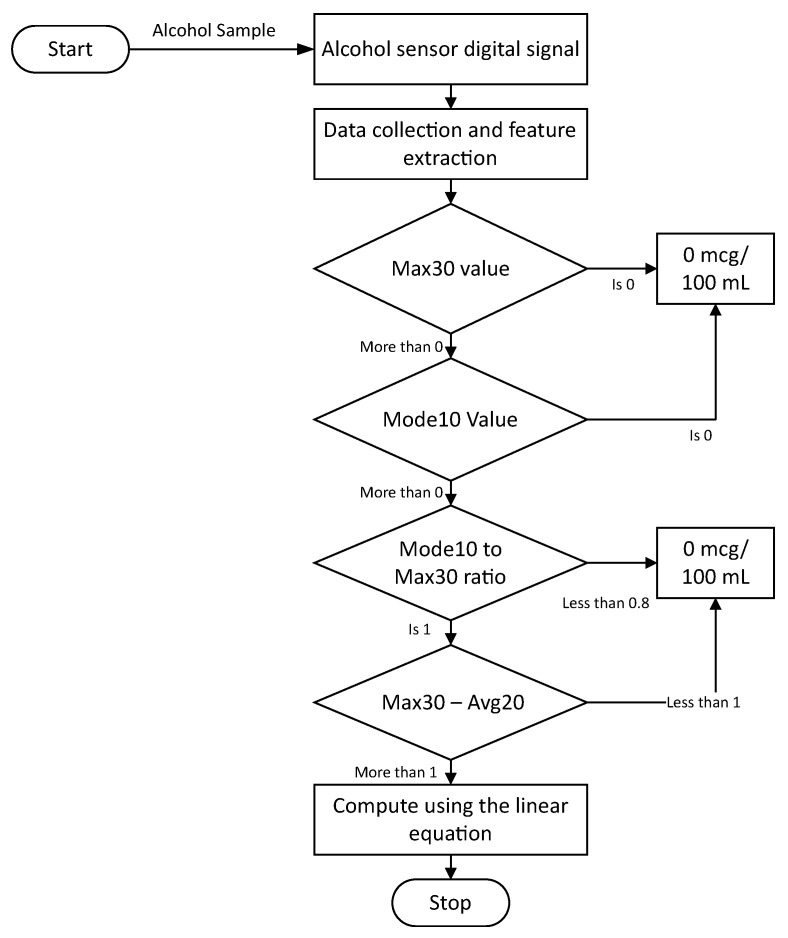
The threshold algorithm for classifying zero concentrations.

The equation displayed in Figure 7 relates the Max30 feature obtained from the sensor with the alcohol concentration in a linear relationship. As shown in Figure 6a, the clean sample still resulted in a slight increase in the sensor’s digital signal; hence, this model may risk computing false positive results at zero concentrations. To avoid this false positive result, an algorithm to classify the zero concentration can be introduced to avoid the usage of the linear equation at what is supposed to be zero concentration. Four logical rules were drawn to design a threshold algorithm based on the manual observation of the collected data. As shown in Figure 8, the first condition after the feature extraction comprises examining the Max30 feature, which represents the peak response; if that feature is equal to zero, then the outcome is obviously zero as no changes occurred relative to the sensor’s digital signal. If the value is greater than zero, then the second condition will be examined, which comprises examining the Mode10 feature that determines the most repeated numbers within the first 10 readings of the sensor; if the value of Mode10 is zero, then the concentration will be computed as zero since no sufficient reaction was present to cause a meaningful change in the sensor’s digital signal. Let us suppose that the value of Mode10 is more than zero. In that case, the third condition will be examined, which examines the relationship between the Mode10 and Max30 features since Mode10 is determined as the most frequently occurring number in the first 10 readings of the digital signal and Max30 represents the peak value of the digital signal. The distance between the two should not be relatively large compared to the sensor’s response when fed with actual alcohol remains in terms of fluctuating near the peak within that area. The chosen ratio for that purpose is 0.8. If the ratio is below 0.8, then a zero concentration will be computed. Suppose that the ratio is greater than 0.8 (which existed in a few readings in the training data set). In that case, the fourth condition will be executed, which examines the difference between Max30 and Avg20 features; if the distance between these features is less than 1, then a zero concentration will be computed. This is because Avg20 looks at the average of the first 20 readings, which are supposed exhibit a slight downtrend trend, and a difference that is less than 1 indicates an insufficient peak that is followed by an actual downtrend and simply exhibits electrical noise. Finally, if the distance value mentioned in the fourth condition is greater than 1, then the linear equation will be utilized to quantify alcohol concentrations.

**Figure 9:**
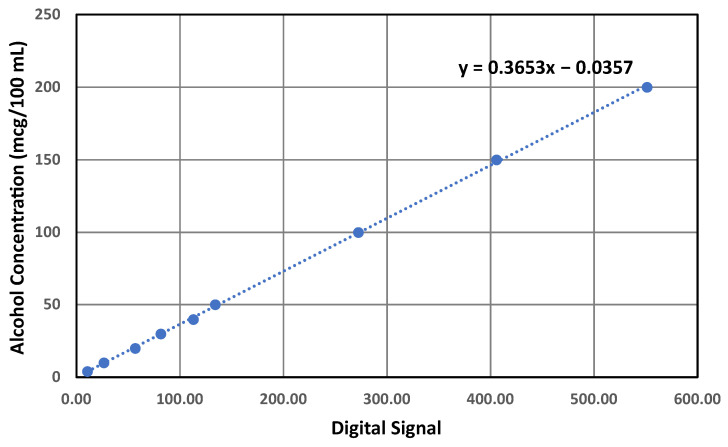
Linear equation obtained from plotting the average peaks of the digital readings (x) against the alcohol concentrations (y) after excluding the zero point.

After excluding the zero concentration from the model, a new linear relationship was plotted to determine the concentration based on the obtained Max30 feature extracted from the sensor’s digital signal. The final linear equation obtained from this method is displayed in Figure 9.

### 3.5. Experimental Setup and Performance Evaluation

Previously collected data (Section 3.2) were randomly split into a training set (90% of the data set) and a validation set (10% of the data set). The extracted features (Section 3.3) from the training set were used when developing the alcohol quantification algorithm. Similarly, the extracted features from the validation set were used to validate the developed algorithm.

Later on, the developed models were uploaded to the microcontroller (ATmega 2560) for experimental evaluation. The concentrations used in this step were 25, 75, 125, and 180 mcg/100 mL.

To evaluate the performance of the quantification algorithm, the following performance metrics were used: (1) mean square error (MSE), (2) root mean square error (RMSE), (3) mean absolute error (MAE), (4) the coefficient of determination $\left(R^{2}\right)$, (5) the accuracy percentage (Accuracy%), (6) the standard deviation, and (7) the relative standard deviation (%RSD), shown in Equations (5)–(11), respectively. The values of $BrAC_{e}$ and $BrAC_{a}$ refer to the predicted BrAC obtained from the algorithm and the actual BrAC, respectively. In contrast, $BrAC_{a.avg}$ represents the average of all actual BrAC values, and the value of $\bar{X}$ refers to the average of the predicted BrAC values at the corresponding actual BrAC.

(5) $$\mathrm{RMSE}=\;\sqrt{\frac{1}{M}\sum_{i=1}^{M}{\left(BrAC_{e}-BrAC_{a}\right)}^{2}}\;\;\;\;\;\;\;\;\;\;\;\left(\frac{\mathrm{mcg}}{100\;\mathrm{mL}}\right)$$

(6) $$\mathrm{MSE}=\frac{1}{M}\sum_{i=1}^{M}{\left(BrAC_{e}-BrAC_{a}\right)}^{2}\;\;\;\;\;\;\;\;\;\;\;\;\;\;\;\;\;\;\;\;\left(\mathrm{mcg}/100\;\mathrm{mL}\right)$$

(7) $$\mathrm{MAE}=\frac{1}{M}\sum_{i=1}^{M}\left|\left(BrAC_{e}-BrAC_{a}\right)\right|\;\;\;\;\;\;\;\;\;\;\;\;\;\;\;\;\;\;\left(\mathrm{mcg}/100\;\mathrm{mL}\right)$$

(8) $$R^{2}=1-\frac{\sum_{i=1}^{M}{\left(BrAC_{a}-BrAC_{e}\right)}^{2}}{\sum_{i=1}^{M}{\left(BrAC_{a}-BrAC_{avg}\right)}^{2}}$$

(9) $$\mathrm{Accuracy}\%=100\%-(\frac{BrAC_{a}-BrAC_{e}}{BrAC_{a}}\times 100\%)$$

(10) $$\mathrm{Standard}\;\mathrm{deviation}\left(\sigma \right)=\;\sqrt{\frac{\sum {\left(BrAC_{e}-\bar{X}\right)}^{2}}{M-1}}\;\;\;\;\;\;\;\;\;\;\;\;\;\;\;\;\;\;\left(\mathrm{mcg}/100\;\mathrm{mL}\right)$$

(11) $$\mathrm{RSD}\%=\frac{\sigma_{r}\;\times \;100}{\bar{X}}$$

### 3.6. IoT Integration

**Figure 10:**
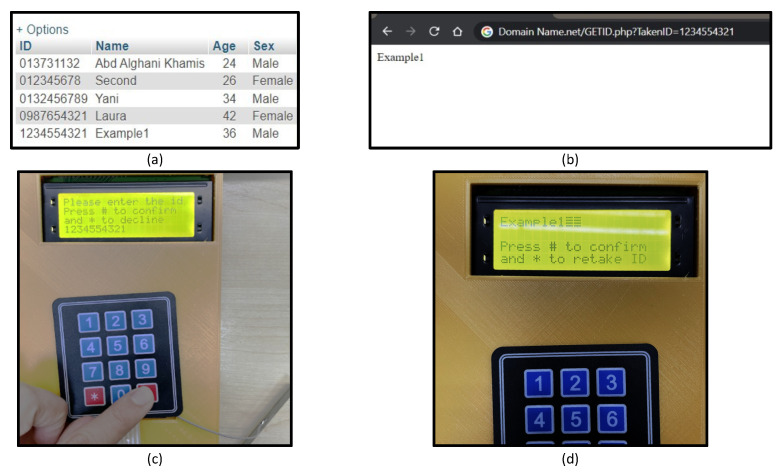
Communication with the table stored in the online database (**a**) through the browser (**b**) and the microcontroller (**c**,**d**).

The chosen module to be integrated with the device was SIM7600CE since it can operate at the frequencies of the local networks. A conditioning board to connect SIM7600CE to the microcontroller board was obtained from DFRobot. The data generated by the device are divided into two parts: the participant’s identification number and the detected alcohol concentration. The HTTP GET request method is used to enable communication with the Internet. The identity (ID) number should be sent in as the first request to obtain the related details from the database, and the BrAC will be sent in the second request in order to be stored in the database. In our prototype, the participant’s name should return to the device for confirmation purposes. A mock online domain was created as an address to access the database. Two tables were created in the database to contain the identification information and the reported values of the device. Two separate hypertext preprocessor (PHP) scripts were created in unique addresses in the cloud base to request and insert data into the database. The GET request includes the data to be processed by the PHP scripts. Figure 3 shows the device’s online communication concept. The microcontroller executes the GET request to obtain the name related to the ID taken, and upon confirmation (as shown in Figure 4), the GET request is executed to upload the detected alcohol concentration together with the corresponding participant ID to the cloud. Figure 10a shows an example of a table that was created in the database to carry ID numbers and related information, Figure 10b shows an example of the result obtained from executing the GET request to retrieve the corresponding name using a browser, Figure 10c shows the screen output and the number pad input before executing the same GET request in the developed device, and Figure 10d shows the response given by the device after receiving the database response. The used commands by the cellular module are presented with examples and expected returns in Appendix A.

## 4. Results

This paper presents a proof-of-concept prototype for an IoT-integrated breath analyzer to quantify breath alcohol concentrations and communicate with an online database via HTTP. Additionally, a performance evaluation of the breath alcohol quantification by several performance metrics was conducted and thoroughly discussed. The following subsection shows the performance evaluation results.

### Performance Evaluation of Alcohol Quantification

The results of the predicted alcohol concentrations using the threshold algorithm based on different performance metrics (Accuracy%, RMSE, MAE, MSE, $R^{2}$, standard deviation, and RSD) on the validation set for the concentrations of 0, 4, 10, 20, 30, 40, 50, 100, 150, and 200 mcg/100 mL, as previously explained (Section 3.5), are presented in Table 3.

From Table 3, it can be seen that the proposed method has successfully predicted the zero concentration with 100% accuracy and no errors or deviations, and this excludes the possibility of a false positive in the absence of ethanol. The accuracy for the remaining concentrations ranges between 96% and 99.37%, with the exception for the concentration at 10 mcg/100 mL, which has the lowest accuracy value of 92.8%. The coefficient of determination for the on-machine validation is 0.9995; however, this value is expected to drop in experimental testing, as these concentrations (0, 4, 10, 20, 30, 40, 50, 100, 150, and 200 mcg/100 mL) are the same concentrations used to create the algorithm, unlike an experimental test that tests the algorithm with concentrations that were unused when developing the algorithm. Additionally, the standard deviation was not consistent among the concentrations, and it showed low values ranging from 0 mcg/100 mL to 1.81 mcg/100 mL, which can be explained by the sample size for the on-machine validation being small (10% of the collected data).

After validation of the algorithm, it was uploaded to the microcontroller for experimental testing verification. The embedded model was tested on alcohol concentration values of 25, 75, 125, and 180 mcg/100 mL, as explained in Section 3.5. The performance of the alcohol quantification algorithm based on the performance metrics (Accuracy%, RMSE, MAE, MSE, $R^{2}$, standard deviation, and RSD) is presented in Table 4. Table 4 shows that the average accuracy obtained at each concentration ranged between 97.65% and 98.64%. The lowest and highest average accuracies were observed at concentrations of 180 mcg/100 mL and 25 mcg/100 mL, respectively. Similarly, the highest and the lowest MSE, MAE, and RMSE values were at concentrations of 180 mcg/100 mL and 25 mcg/100 mL, respectively. Additionally, the standard deviation increased as the concentration increased, showing a positive correlation with the actual concentration. This can be confirmed with the RSD, which possesses a close range of values from 1.41% to 2.69%.

**Figure 11:**
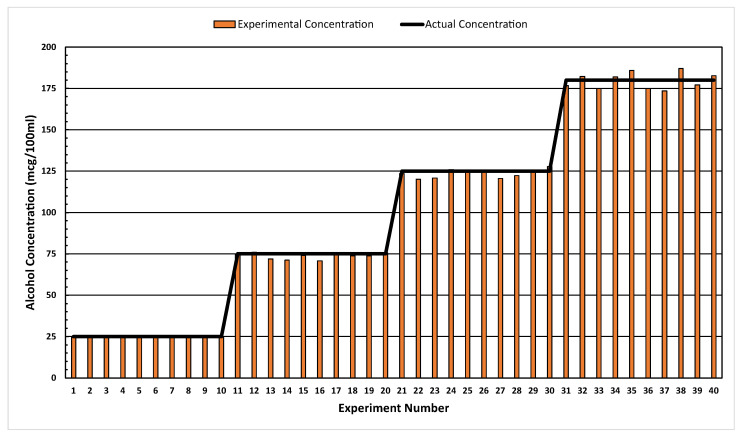
Experimental vs. actual alcohol concentrations at 25, 75, 125, and 180 mcg/100 mL.

Figure 11 shows the results obtained from the experimental validation; the alcohol concentration quantification algorithm produced values close to the actual concentrations for nearly all tests. Additionally, it can be seen that the quantification of alcohol at high concentrations has higher positive errors compared to those of lower concentrations.

## 5. Discussion

In this paper, a conceptual design and a developed prototype of an IoT-integrated breath analyzer for quantifying alcohol concentrations in breath was presented. The implementation of the IoT in the breath analyzer came from the fact that an automated data acquisition and reporting process surpasses manual processes in many aspects, including the elimination of human error [19,20]. When healthcare professionals utilize an IoT-integrated breath analyzer, instantly retrieving a piece of information associated with the participants via an Internet connection is possible. In the proposed prototype, the name associated with a preassigned ID number can be retrieved from the database; however, this concept applies to any other required information as long as the information is available in the database and the device is programmed for specific requirements. Additionally, the breath analysis results can be stored in the database with the relevant information automatically upon confirmation from the user. This concept eliminates human error in the manual method. In contrast, a manual record of information, a manual record of breath analysis results, and a manual report directed toward a database could involve potential errors.

Although several studies were found to implement IoT with an alcohol detection technology [41,45,46,51], this paper presents a concept for implementing cellular IoT with a breath analyzer equipped with input (number pad) and output (LCD display) devices to enable using the device for collecting data from multiple participants.

The breath analyzer in this paper quantifies alcohol concentrations in human breath by using a fuel-cell-based sensing element. The motivation for quantifying alcohol concentrations in human breath came from the possibility of correlating the concentration of alcohol in the breath to that in the blood via a ratio [29,30], although the ratio may differ between healthcare agencies depending on the standards followed by the corresponding country.

To calibrate an alcohol sensor, its signal must be recorded based on the calibration points chosen, and then an equation (regression equation) to relate the change of the digital signal to the concentration of alcohol must be created [63]. The quantification of alcohol concentration in breath was performed by applying a scientific concept to translate the change in the sensor’s digital signals into the amount of alcohol in the breath sample. Observing the characteristics of the sensor signal under different concentrations was the main component of the calibration procedure. In addition to the linear relationship between the peak of the sensor’s digital signal and concentration, other features were used to create multiple thresholds to classify the zero concentrations since a slight increase above the baseline in the digital signals remained despite no alcohol being present in the sample. That slight increase in the digital signal may be explained by either electrical noise caused by the electrical circuits or the actuation of the pump, which may affect the balance of the initial partial pressures of reactants and products in the fuel cell, as explained in the Nernst equation (Section 2.1). The range of the sensor can detect up to 300 mcg/100 mL BrAC; however, the maximum concentration used in the training data set was 200 mcg/100 mL BrAC (which corresponds to 460 mg/100 mL BAC using the BBR of 2300 ) because BrAC values exceeding that may involve alcohol poisoning, potentially fatal conditions, and comas [75,76].

Table 3 presented the performance of the developed algorithm when validating it before uploading it into the microcontroller. The value of $R^{2}$ was 0.9995, corresponding to 99.95% of the quantified concentrations being represented with the regression line; in other words, $R^{2}$ is a quantification of how near each point of data fits the regression line. Despite the $R^{2}$ value, few points were still considered as outliers; the outliers would greatly affect the performance of the linear equation, and that was demonstrated at 10 mcg/100 mL concentration, as it showed a lower accuracy than other concentrations due to not removing the outliers when splitting the data initially. Although the average accuracy of the on-machine validation was high (97.71%), that particular data set did not provide sufficient evidence due to its small size. A sample size of 50 samples may provide sufficient evidence for regression analyses [77]; therefore, the experimental validation set of data may be a better representative of the performance of the alcohol quantification algorithm.

**Figure 12:**
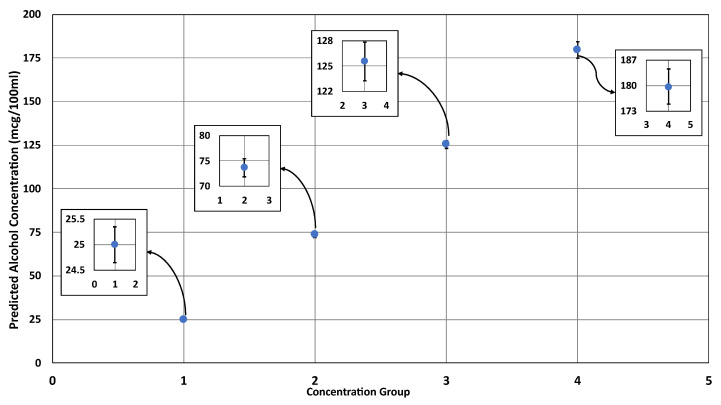
The predicted alcohol concentrations with their standard deviations in concentration groups 1 (25 mcg/100 mL), 2 (75 mcg/100 mL), 3 (125 mcg/100 mL), and 4 (180 mcg/100 mL).

The performance of the quantification algorithm was shown to have a lower $R^{2}$ value for the experimental validation compared to the on-machine validation set, which can be explained by the concentrations used for the experimental validation being unobserved by the regression line. However, the linear regression process is good at predicting a variable based on another variable with similar linear relationships [78], which can be demonstrated with the average accuracy remaining high and close to that of the on-machine validation set. The performance of alcohol quantification showed a consistent trend in their error metrics; it was observed that the values of these metrics increased as the concentration did. Similarly, the increment of the standard deviation of the predicted values was associated with the increment of the actual concentrations. Figure 12 demonstrates the error bars of each concentration group in the experimental validation set. The RSD can explain the association between the increment of standard deviation and the concentration. Relatively, the standard deviations are similar (within 1.41% to 2.60% of average predicted concentration), indicating consistent performance across the concentrations. However, individually considering each concentration’s deviation may be necessary if the applications require high precision, such as law enforcement devices [29].

The theoretical maximum coulombic efficiency of the fuel cell used in the breathalyzer (proton exchange membrane fuel cell) is expected to be within the range of 30–60% [69,79,80,81]. As previously explained in Section 2.1, the oxidation reaction of ethanol on Pt produces four electrons (Reaction 2), and the reaction can be used to determine the circuit’s potential in volts by using the Nernst equation (Equation (4)), where the number of transferred electrons is four and partial pressures of the reactants and products vary according to the different considerations fed into the cell. The partial pressure due to the ethanol concentration is directly proportional to the electrons transferred at the anode, which determines the overall electrical potential. The partial pressure estimation requires further data collection that includes the measurement of the residuals of water and acetic acid [82]; after computing the circuit’s potential, one must account for the losses that occur before proceeding with calculating the coulombic efficiency, which includes the ohmic losses and initiation losses. Finally, the theoretical electrical potential can be compared against the readings obtained from the microcontroller after adjusting for the corresponding voltage.

Although the performance of the fuel-cell sensors was reported as superior compared to other technologies, such as semiconducting sensors [65], readings drift over time and may result in less accurate readings. The degradation of electrodes in the fuel cell can explain this drift [82,83]. Since platinum is a major cause of the oxidation reaction of the ethanol particles in the anode compartment, the degradation of the electrodes would result in a drift in the readings. From the Nernst equation, a positive drift (higher electrical potential than expected) indicates a greater value of partial pressure of reactants than that of products. In contrast, a negative drift (lower electrical potential than expected) indicates a greater value of the partial pressure of products than of reactants. However, when the concentration and the sample size are constant, there are no differences in the actual partial pressure of the reactants when initially fed to the anode compartment, as the same amount of ethanol will be present in a given sample despite any degradation changes in the electrodes. The shift in partial pressures may be explained by different means; as Pt starts to degrade, the reaction may be slower or less efficient, which changes the ratios of elements in the products and reactants. Consequently, the fuel cell would require higher concentrations of the reactants to keep producing the same amount of electrical potential. Setting a frequent recalibration procedure to correct this drift would eliminate the errors resulting from the degradation of the electrodes. However, as continuous drifts and corrections would compromise the detectable range of the cell (smaller maximum detectable concentration or greater minimum detectable concentration), there should be a limited number of recalibrations implemented before the eventual replacement of the cell. The fuel cell used in this work is expected to perform steadily for 6–12 months depending on the frequency of use and tolerance for error. For instance, evidential devices require shorter calibration intervals than low-cost consumer devices [63].

## 6. Conclusions

This paper presents a development methodology and a performance evaluation of an IoT-integrated breath analyzer. Cellular technology was utilized as a gateway for IoT integration to enable country-wide coverage. The Internet protocol (HTTP) employed and the procedure for sending and retrieving data between the device and a cloud-based database were discussed. The modules used in the device for each function were demonstrated and described.

The automatic data collection and reporting method may allow healthcare representatives to identify the alcohol consumption patterns and the associated influencing factors for each community. An algorithm quantified alcohol in breath based on a threshold and linear regression concept. The experimental validation of the alcohol quantification algorithm shows that the performance remains similar despite experimenting with different concentrations from those used in the algorithm’s development process.

The practical implication of this work is that an IoT-equipped breath analyzer provides a method that enables instant reporting of the collected breath alcohol concentrations in a cloud-based database, resulting in conserved efforts and eliminating errors in human reporting. This is particularly helpful in scenarios where a large-scale study of a community requires multiple alcohol measurements to be carried out. The social implication of this work is highlighted when a community’s regular access to healthcare representatives remains a challenge.

## 7. Outlook

The following recommendations may provide aid in future research studies and provide enhancements:

1. The developed device implemented one type of sensing technology (fuel cell). Therefore, future work may investigate different sensing technologies.
2. The IoT integration in this work was performed by utilizing cellular technology to enable a wide range of coverage (throughout the country within the local cellular operators’ service areas); however, the energy consumption of this technology was not investigated. Hence, future work may study this aspect when implementing cellular IoT in such applications.
3. The Internet protocol used in this work is the HTTP using the GET request due to the small size of data to be transferred between the device and the cloud that does not require a complex protocol to account for connection times; however, HTTP GET may become a limitation when larger applications with larger sets of data need to be sent for each request. Therefore, future research comparing the performances of different protocols in terms of speed in the cellular IoT scope may provide beneficial insights and guidelines for choosing a proper protocol with the cellular IoT.
4. The quantification algorithm presented in this research was manually developed; future research may investigate other methods for algorithmic developments, such as machine learning.

## Figures and Tables

**Table 1:** Comparison between different types of alcohol detection technologies.

Technology	Criteria				
	Portability	Online Connectivity	Wide-Scale Usability	Continuous Monitoring	Independence
Wearable devices	x	x		x	x
Regular breath analyzers	x		x		
Personalized IoT-integrated breath analyzers	x	x			
Vehicle-integrated device		x			x
Indirect detection			x		
Proposed solution (cellular IoT breath analyzer)	x	x	x		x

**Table 2:** The frequencies and interface standards provided by local operators in Malaysia [72].

Frequency	Interface	Operators
900 (E-GSM)	GSM	Celcom, Digi, Maxis, U-mobile
1800 (DCS)	GSM	Celcom, Digi, Maxis, U-mobile
B1 (2100)	UMTS	Celcom, Digi, Maxis, U-mobile
B8 (900 GSM)	UMTS	Maxis
B3 (1800+)	LTE	Celcom, Maxis
B7 (2600)	LTE	Celcom, Digi, Maxis, U-mobile

**Table 3:** Results of the on-machine validation of the alcohol quantification algorithm.

Actual Concentration (mcg/100 mL)	Average Predicted Concentration (mcg/100 mL)	Performance Metrics						
		Average Accuracy (%)	MSE (mcg/100 mL)	MAE (mcg/100 mL)	RMSE (mcg/100 mL)	R^2^	Standard Deviation (mcg/100 mL)	RSD (%)
0	0	100	0	0	0	-	0	0
4	3.98	99.5	0	0.02	0	-	0	0
10	9.28	92.8	0.55	0.72	0.74	-	0.25	2.69
20	19.69	98.45	0.1	0.31	0.32	-	0	0
30	29.01	96.68	1.83	1	1.35	-	1.29	4.45
40	39.9	96.8	2.77	1.28	1.66	-	1.81	4.41
50	49.96	96.34	3.87	1.83	1.97	-	0.57	1.16
100	99.97	98.6	2.52	1.41	1.59	-	1.04	1.05
150	150.15	98.61	5.58	2.09	2.36	-	1	0.68
200	199.51	99.37	1.63	1.28	1.28	-	1.8	0.9
Overall		97.71	1.88	0.99	1.37	0.9995	-	-

**Table 4:** Results of the experimental validation of the alcohol quantification algorithm.

Actual Concentration (mcg/100 mL)	Average Predicted Concentration (mcg/100 mL)	Performance Metrics						
		Average Accuracy (%)	MSE (mcg/100 mL)	MAE (mcg/100 mL)	RMSE (mcg/100 mL)	R2	Standard Deviation (mcg/100 mL)	RSD (%)
25	24.8	98.64	0.15	0.34	0.39	-	0.35	1.41
75	73.68	97.78	4.67	1.67	2.16	-	1.81	2.46
125	125.55	98.58	4.93	1.77	2.22	-	2.27	1.81
180	179.69	97.65	21.16	4.24	4.6	-	4.84	2.69
Overall		97.71	7.73	1.32	2.78	0.9977	-	-

## Data Availability

Not applicable.

## Abbreviations

IoT	Internet of Things
HTTP	Hypertext transfer protocol
PHP	Hypertext preprocessor
GSM	Global System for Mobile communication
BrAC	Breath alcohol content
BAC	Blood alcohol content
BBR	Breath alcohol to blood alcohol ratio
mcg/100 mL	Micrograms of alcohol per 100 milliliters of breath
Pt	Platinum
${\mathrm{CH}}_{3}{\mathrm{CH}}_{2}\mathrm{OH}$	Chemical formula of ethanol
${\mathrm{CH}}_{3}\mathrm{CHO}$	Chemical formula of acetaldehyde
H	Hydrogen
$e^{-}$	Electron
$H_{2}O$	Chemical formula of water
${\mathrm{CH}}_{3}\mathrm{COOH}$	Chemical formula of acetic acid
${\mathrm{CO}}_{2}$	Chemical formula of carbon dioxide
OCP	Open circuit potential
E	Thermodynamic voltage
$E_{o}$	Reversible voltage standard
*T*	Temperature
*R*	Ideal gas constant
*n*	Number of transferred electrons
*F*	Faraday constant
$P_{r}$	Partial pressure of the reactants
$P_{p}$	Partial pressure of the products
2G	Second generation of cellular network
3G	Third generation of cellular network
4G	Fourth generation of cellular network
LTE	Long-term evolution
Mbps	Megabits per second
kbps	Kilobits per second
mL	Milliliter
A/D	Analog-to-digital
V	Volts
mv	Millivolts
ms	Milliseconds
MSE	Mean squared error
RMSE	Root mean squared error
MAE	Mean absolute error
$R^{2}$	Coefficient of determination
RSD	Relative standard deviation
σ	Standard deviation
$BrAC_{e}$	Predicted BrAC obtained from the algorithm
$BrAC_{a}$	Actual BrAC obtained from the algorithm
$BrAC_{a.avg}$	Average of all the actual BrAC values
$\bar{X}$	Average of the predicted BrAC values at a certain concentration

## Appendix A

The commands used in the SIM7600 module to initiate and execute the HTTP GET requests are presented in Table A1.

**Table A1:** Commands used to initiate HTTP in the cellular module.

Command	Purpose	Expected Responses and Interpretation
AT	Checking the readiness of the chip	OK, system is ready
		ERROR, wiring issue
AT+HTTPTERM	Termination of HTTP	OK, protocol has been terminated
		ERROR, protocol is already terminated
AT+HTTPINIT	Initialization of HTTP	OK, protocol has been initialized
		ERROR, protocol cannot be initialized
AT+HTTPPARA =	Sending HTTP parameters: should include the URL for the website (e.g., AT+HTTPPARA = URL, DomainName.net/GETID.php?TakenID=1234554321”)	OK, address is correct, and the status is online
		ERROR, incorrect address
AT+HTTPACTION = 0	Choosing HTTP GET method	+HTTPACTION: 0, 200, 18, HTTP GET is ready and 8 is the length of the content
		ERROR, Server error
AT+HTTPREAD = 0, 18	Reading the content from position 0 to position 18, this code is based on the previous output	Displaying the name, (e.g., Example 1)
		A response from the cloud: “No ID found”

An example of an HTTP GET request that can be sent to the cellular module by the microcontroller for uploading an ID to the cloud and retrieving a name is “AT+HTTPPARA = URL, https://DomainName.net/GETID.php?TakenID=IC”, where AT+HTTPPARA is the command used between the microcontroller and cellular module that carries the parameters for the HTTP request; URL stands for Uniform Resource Locator, which is used in the World Wide Web to locate domain addresses; GETID.php is the PHP script that must be pre-uploaded to the cloud and used to retrieve the corresponding information (in this application, the name of the participant) relative to the ID number given by the full HTTP GET request and to provide the appropriate response; TakenID is the variable used in the PHP script to read the ID number given in the request; and IC is the variable used by the microcontroller to carry the ID number. An example of an HTTP GET request for uploading breath alcohol concentrations together with the corresponding ID number to the cloud is “https://DomainName.net/Insert.php?TakenID=IC&BrAC=BAC”, where Insert.php is the PHP script that must be pre-uploaded to the cloud and used to create a new record of the information given by the full HTTP GET request and to provide the appropriate response (whether it succeeded or not); TakenID is the variable used in the PHP script to read the ID number given in the request; BrAC is the variable used in the PHP script to read the breath alcohol concentration given in the request; IC is the variable used by the microcontroller to carry the ID number; and BACV is the variable used by the microcontroller to carry the breath alcohol concentration that was computed previously.
